# The Challenges and Value of Using Video-Assisted Education Tools and Digital Information Systems for Post-discharge Care in Elderly Patients: A Scoping Review

**DOI:** 10.7759/cureus.86319

**Published:** 2025-06-18

**Authors:** Monish Hassan, Asif Iqbal, Giovanni Brambilla, Fathima Almas, Deepak V Pathiyil, Jyothi Janardhanan Kakkra

**Affiliations:** 1 Internal Medicine, Mediclinic City Hospital, Dubai , ARE; 2 Infectious Disease, Mediclinic City Hospital, Dubai, ARE; 3 Internal Medicine, Mediclinic City Hospital, Dubai, ARE; 4 Radiation Oncology, American Hospital Dubai, Dubai, ARE; 5 Infectious Disease, University of Cambridge, Cambridge University Hospitals NHS Foundation Trust, Cambridge, GBR; 6 General Surgery, West Suffolk NHS Foundation Trust, Bury, GBR

**Keywords:** digital information systems, discharge education, geriartric discharge summary, hospital-based education, patient education, video discharge education

## Abstract

Continuity of care plays a vital role in ensuring the well-being of elderly patients as they transition from hospital to home. In recent years, video-assisted and digital information systems have emerged as potential tools to support this critical phase. While these technologies hold promise, there has been limited examination of their actual impact on patient outcomes and the experiences of patients and caregivers. To better understand their effectiveness, a scoping review was conducted to evaluate how well these systems maintain continuity of care and how they are perceived in post-discharge settings. The review adhered to established scoping methodologies and focused on English-language studies published between 2010 and 2022. Sources include Medline, Google Scholar, and Web of Science. Studies featuring video-based or digital discharge information, regardless of patients’ age, were included to gain insight into implementation challenges. In total, 23 studies met the inclusion criteria.

This review reveals that in certain settings, video-assisted discharge education systems can improve patient recall, reduce hospital readmissions and revisit rates, and are generally viewed positively by patients and caregivers, particularly when the information is tailored to the individual. However, several obstacles to effective implementation were also identified, including patients’ discomfort with technology, resistance from healthcare providers, social barriers, and the time constraints associated with rapid discharge processes. Despite these challenges, video-assisted and digital discharge education systems show considerable potential to enhance post-discharge care. Their effectiveness, however, depends on various factors such as patient digital literacy, the clarity and personalization of the content, and the strength of the implementation process. To fully realize their benefits, it will be essential to address technological discomfort and provider resistance, ensuring that these tools are smoothly integrated into transitional care practices.

## Introduction and background

The introduction of the diagnosis-related groups (DRG) system for insurance reimbursement has significantly reshaped priorities within healthcare organizations. By disconnecting financial incentives from the length of hospital stays, the system has prompted large healthcare corporations to minimize inpatient durations. This shift has created a pressing need to deliver comprehensive medical care during hospitalization and ensure continuity of care following discharge, particularly in the home setting. One major consequence of insufficient post-discharge information has been the frequent readmission of patients, which continues to burden healthcare systems. However, the COVID-19 pandemic has contributed to a broader acceptance of telemedicine, as patients have become more open to technology-driven healthcare solutions. This growing comfort with digital tools presents a valuable opportunity to reimagine how discharge care is delivered.

At the same time, demographic changes are transforming the landscape of healthcare. With global populations expanding and life expectancy increasing, people are living longer and requiring more complex, long-term medical support. These evolving needs call for more adaptable and effective healthcare communication strategies. Research has consistently shown that individuals respond better to visual and auditory information compared to written text [1]. Strategies such as medication reconciliation, structured discharge communication, and targeted education for patients and families have all been identified as crucial components for improving continuity of care. These practices help reduce adverse events, enhance patient safety, and support smoother transitions from hospital to home.

Despite these insights, a considerable gap remains in how discharge information is communicated. This gap underscores the urgent need for improved discharge communication strategies, which can only be achieved through coordinated efforts among all stakeholders involved in the discharge process. By enhancing communication practices, healthcare providers can better meet patient needs and expectations. In response to these challenges, the implementation of a digital and video-assisted discharge information system is proposed as a way to improve patient satisfaction, especially among older adults, and to reduce readmission rates. This scoping review is designed to evaluate the effectiveness of digital information systems in delivering discharge information. It also seeks to explore the limitations and challenges of these systems from the perspectives of patients, caregivers, and healthcare professionals. The insights gained will support the development of a more effective and patient-centered discharge summary process through the use of digital technologies.

## Review

This review unearths the existing evidence on the value and efficiency of video-assisted and digital information systems in maintaining continuity of care in elderly patients and the post-discharge care of elderly patients from the perspectives of patients and carers. It also discusses the challenges related to its use both by patients and healthcare workers.

Methodology

We applied the standard framework reported by Arksey and O’Malley and further extended by the Joanna Briggs Institute [2-5]. We also adapted the reporting guidelines described in the Preferred Reporting Items for Systematic Reviews and Meta-analyses Statement (PRISMA), an extension of PRISMA for scoping reviews (ScR) [6]. Ethical approval was not required for this review, as it involved analysis of published data.

Inclusion Criteria

The inclusion criteria for this scoping review were determined using the 'population, context, and concept' (PCC) framework [5]. The population considered includes patients being discharged from healthcare settings who have received video-assisted or digital discharge information. No specific demographic group was excluded, allowing for a broad inclusion that supports the objectives of a scoping study. This decision was rooted in prior research that highlighted certain parallels between pediatric and geriatric populations, particularly regarding aspects of comprehension and the necessity for assisted care [7,8]. However, recognizing the inherent differences between these distinct age cohorts, only those pediatric studies that demonstrated clear potential for reproducibility and applicability within the geriatric age group were selected. This selective approach was a pragmatic necessity, driven by the limited availability of research focused exclusively on geriatric populations.

Search Strategy

The search strategy aimed to identify both published and unpublished literature, beginning with an initial scan of databases including MEDLINE, Google Scholar, and Web of Science. Terms found in the titles, abstracts, and indexing of relevant articles informed a comprehensive search strategy. The scoping review focused on the broad concept of video-assisted discharge education systems and other digital information systems. The primary context for this investigation was to map the existing literature related to their potential for improving patient outcomes and satisfaction following discharge. The research team agreed upon the terms for the search, and one author conducted the searches independently based on those terms before aggregating the sources for all authors’ reviews. Searches across all databases included synonyms and terminological variations with Boolean operators to broaden the results. All identified keywords and indexing terms were incorporated, and reference lists of relevant articles were also reviewed to identify additional sources. The review focused on studies published between 2010 and 2022.

Source of Evidence Selection

In terms of evidence sources, both qualitative and quantitative research designs were considered, as well as opinion pieces, systematic reviews, case-control studies, and cohort studies. Following the literature search, all references were organized and uploaded into EndNote X9.2 (Clarivate, Philadelphia, PA, USA) for deduplication. A single reviewer then assessed all citations to determine whether they met the inclusion criteria. This was consequently reviewed by another reviewer. Studies excluded during this process were documented with reasons for their exclusion. Final selections were reported using the PRISMA-ScR flow diagram [6]. 

Data Extraction

To extract and organize data consistently, a standardized data extraction form was used. This form captured details such as the article’s authorship, publication year, journal or source, country of origin, objectives, methodology, interventions described, and findings relevant to the scoping review. A pilot extraction was initially conducted to ensure the form’s comprehensiveness. 

Analysis of Evidence

**Figure 1:**
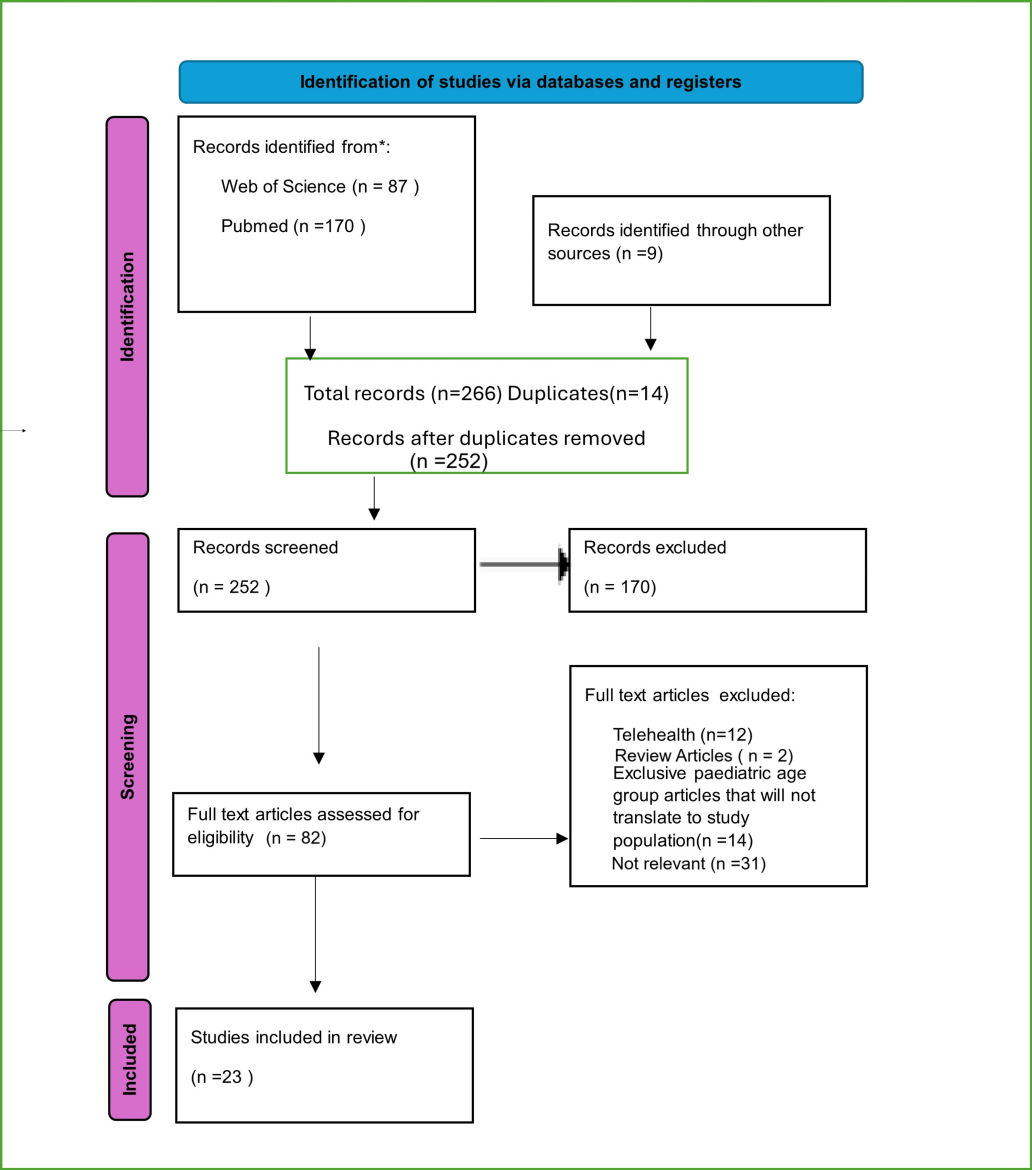
The PRISMA-ScR was used for data extraction and identification of relevant articles PRISMA-ScR: Preferred Reporting Items for Systematic Reviews and Meta-Analyses extension for scoping reviews

The data were analyzed using the PRISMA-ScR framework (Figure 1) [6]. Articles were reviewed and coded into themes based on their relevance to the research question. Thematic analysis was used to distill the key findings and organize them into a cohesive narrative. This analysis aimed to highlight major trends and draw meaningful conclusions from the literature. The results were then presented in a thematic narrative supported by diagrams, figures, and tables where applicable. The initial database search yielded 252 articles. After removing duplicates and screening titles and abstracts, 170 articles were excluded. The remaining 82 were reviewed in full, leading to the exclusion of an additional 59 articles. Twenty-three articles were included in the review, of which one was specific to the pediatric age group. All six researchers agreed on its inclusion. Due to the inherent heterogeneity across included study designs, populations, and reported outcomes, a meta-analysis was not feasible. Consequently, findings were synthesized narratively, organized by emergent thematic domains. No statistical tests or effect estimates were calculated.

Results

Themes

In this scoping review, a thematic analysis approach was employed to organize and interpret the findings from the literature, allowing for a more structured and meaningful synthesis of diverse evidence (Table 1). The use of themes enabled the reviewers to identify patterns, trends, and relationships across a broad range of studies, which in turn helped address the key review questions more effectively. This thematic analysis helped reveal the existing evidence on the value and efficiency of video-assisted and digital information systems to maintain continuity of care in elderly patients.

**Table 1:** Characteristics of studies identified by PRISMA-ScR and analysed for scoping review PRISMA-ScR: Preferred Reporting Items for Systematic Reviews and Meta-Analyses extension for scoping reviews

Authors	Year	Study type	Country	Objective	Intervention	Type of discharge summary	Findings	Limitations
Wang YC et al. [9]	2019	Case control	Taiwan	Effectiveness of timely discharge plan for long term care patients	Earlier discharge plan	Not mentioned	Reduced rates of emergency department revisit (2.4 vs. 4.8) reduced rate of readmission within 30 days (11.9 vs. 23.8)	Unclear type of discharge summary
Pimentel C et al. [10]	2019	Evaluation of programme	USA	Telehealth vs. inpatient consultation in veterans of geriatric age group	Telehealth to reduce travel time and difficulties associated with the same	Discharge summaries are not video assisted	Video modality used to confer with patients shows positive net result wherein they are comfortable with use of the same	Veterans are more technologically inclined compared to the general population
Wong E et al. [11]	2021	Multistage design qualitative enquiry	China	Post discharge information summary evaluation	Post discharge information summary	Comprehensive	Doctors are deterrents in pilot run; busy schedule may impede preparing comprehensive discharge plan	Study design only
Siddiqui T et al [12]	2020	Mixed method study	Norway	Mini mental state examination (MMSE) vs. ability to understand discharge instruction by assessing response	Video discharge analysis	Discharge explained in detail	Cognitive ability of patients checked; reduced MMSE score impaired understanding of discharge instruction	Did not correct the same; need to provide information to caregivers/ relatives
Steel PA et al. [13]	2021	Intervention analysis	USA	Smartphone based access to patient discharge information	Smartphone based discharge instructions	Discharge instructions that are easily accessible to patients	Reduced emergency department visits; improved patient satisfaction	Restricted to emergency department
Heyworth et al. [14]	2014	Pilot study, survey	USA	Website: Patient web portal in veterans for medication reconciliation	Secure messaging for medication reconciliation tool (SMMRT)	Ambulatory medication reconciliation tool	Patients enthusiastic; 90% will use SMMRT again	Patients used self verification; no face to face verification was required and this may have led to missed discrepancies
Atzema et al. [15]	2013	Prospective, single- centre randomized controlled trial	Canada	Effect of viewing an online video of diagnosis: Specific discharge instructions on patient comprehension and recall of instructions	Online video discharge	Online diagnosis specific video discharge	Improved comprehension in video group	Fewer patients of low socioeconomic status as only single centre used; educational level of patients not assessed
Cece et al. [16]	2021	Randomised controlled, non blinded trial	USA	Compare efficacy and patient satisfaction of video discharge instructions in neonates for first time parents	Video instructions	Video discharge	No statistical difference in terms of overall satisfaction; non statistical increase in confidence in intervention group	Small sample size
Emery et al. [17]	2015	Prospective randomized controlled pilot study	USA	Determine differences in clinical outcomes between voiceover interactive power point (VOIPP) catheter care education vs. usual education in catheter care knowledge	VOIPP plus DVD	VOIPP along with usual care education	No statistical significant change in catheter-related blood stream infections; more calls in VOIPP group	Small sample size
Alexander et al. [18]	2021	Descriptive survey	Australia	Evaluate patient preferences for using technology in communication about symptoms post hospital discharge	Nil	Not specified	Older patients have less preference for technology, patients with issues of minimal concern and patients of high income prefer using technology	Small sample size; many samples lost to follow up
Burgdorf et al. [19]	2022	Multisite qualitative study using semi structured interview	USA	Identify barriers and facilitators to family caregiver training during home healthcare	Nil	Not specified	Educational materials not user friendly; background information brief and inaccurate	Results may not be transferable
Shuen et al. [20]	2018	Single site randomised controlled trial design pilot study	USA	Comparing telephone, texted or typed out post discharge instructions and follow-up	Telephone call back within 24 hours in phone call arm; text message within 48 hours in text message arm	Text and phone call in addition to printed discharge summaries	Reduced number of ED revisits but not statistically significant; no difference in patient satisfaction	Patients on call back asked for the primary physician for clarification. This was noted more often in the phone group than the text group.
Marvel et al. [21]	2021	Multiphase, multicenter, non-randomized controlled trial	USA	Digital health intervention in acute myocardial infarction to reduce all cause readmissions	Corrie health digital platform, Corrie app, Apple watch , I health wireless BP monitor	Digital discharge and follow up with help of devices and health platform	Risk of all cause readmission within 30 days post discharge was 52% lower in intervention group, the perceived app usability was 57.6 and ranged from 30 to 82.5	The proportion of patients in the intervention group were much lesser than the number of patients who had a myocardial infarction due to stringent inclusion criteria
Hoek et al. [22]	2021	Large multicentre randomised control trial	Netherlands	Video discharge instructions in the ED to reduce post-concussion symptoms after three months in patients with mild traumatic brain injury	Video discharge instructions	Video discharge	The Rivermead post concussion questionnaire showed no difference in control and intervention group; anxiety and depression were slightly lower in intervention group, no difference in recall and quality of life	Selection bias
Lee et al. [23]	2018	Randomized controlled open label	South Korea	Evaluate the effectiveness, reproducibility and durability of tailored mobile coaching (TMC) on diabetes management	Tailored mobile coaching	Tailored mobile coaching	Six month assessment of significant reduction in HbA1c by 0.6% in phase 1 and phase 2 trials	Some of the changes may be attributed to change in antidiabetic medication
Rosner et al. [24]	2017	Multicentre observational cohort study	USA	Impact of automated digital patient engagement (DPE) platforms on potentially avoidable costs, hospital admissions, complications post discharge follow-up on hip and knee arthroplasties	Digital patient engagement platform	Digital patient engagement platform	Higher proportion of patients enrolled in DPE had 0 cost in 90 days. Around 45.4% relative reduction in hospital admissions that was not significant, and 54.5% relative reduction in complications.	Patients admitted to the hospital for reasons other than complications post procedure were also included and may have overestimated the number of readmissions in the intervention group
Paruchuri et al. [25]	2021	Open label single arm multicentre clinical trial	USA	Study outcomes of a smartphone based application with live health coaching post percutaneous coronary intervention	Smartphone based application with live health coaching	Digital health platform	Increase in enrolment in cardiac rehabilitation in intervention group, patients are willing to engage in an app targeting cardiovascular disease	Required smartphone usership; participants who opt to enrol may be more engaged
Newnham et al. [26]	2014	Descriptive pilot study	Australia	Feasibility of personalised interdisciplinary audiovisual summary to facilitate care transfer at discharge	Audiovisual record of a Care TV filmed at patient’s bedside by a consultant led interdisciplinary team	Audovisual-aided discharge	Simple to implement; patient recall was satisfactory and easy to use	Larger study required
Eren et al. [27]	2022	Prospective randomised control trial	Turkey	Effect of video-assisted discharge education after total hip replacement surgery	Video-assisted discharge education (VADE)	VADE	No statistically significant difference in pain level. Patient satisfaction, and movement scores were better in the VADE group.	Single centre only
Bloch et al. [28]	2013	Randomised controlled trial	USA	Effect of video discharge instructions in addition to standard written instructions for caregivers’ understanding of child's care plan	Video discharge instructions	Video discharge plus conventional written discharge	Caregiver knowledge and caregiver satisfaction were both improved in the intervention group	Limited to English speaking caregivers
Sinha et al. [29]	2019	Single arm feasibility pilot study	USA	Efficiency of video education intervention to improve patient self efficacy surrounding discharge medication barriers	Video education tool	Video education	Majority of nurses believed the intervention saved time during discharge; barriers were noted to be technological issues. Patients noted confidence increase and knowledge retention; patients were able to tackle medication barriers post discharge.	The video education intervention may only work with patients of specific profiles
Okoniewska et al. [30]	2015	Qualitative analysis	Canada	To analyse perceptions of healthcare workers of barriers to discharge planning	No intervention	Not specific	Communication, role clarity, lack of resources	_
Richmond et al. [31]	2021	Quality improvement analysis	Wales	Quality improvement in discharge summaries	Kotter’s eight-step model used; 'plan, do, study, act' used to overcome barriers	Conventional discharge done early	Model of change used was successful	Used in conventional summaries

Readmission, Revisit, and Complication Rates

Evaluating readmission and revisit rates is an important approach to understanding whether video-assisted and digital discharge interventions lead to meaningful clinical outcomes for patients. These metrics serve as indicators of the effectiveness of discharge processes and the quality of post-discharge care. Evidence indicates that early discharge planning, particularly in elderly patients, can significantly reduce both emergency department revisits and 30-day readmission rates. One study reported that such planning lowered emergency department revisits from 4.8% to 2.4% and reduced 30-day readmissions from 23.8% to 11.9% [9]. These findings are consistent with the outcomes observed in studies employing video-assisted and digital discharge summaries. For instance, one study demonstrated that delivering discharge information through smartphones in emergency department settings significantly reduced unscheduled return visits at 72 hours, nine days, and 30 days [19]. Similarly, a large multicentre trial assessing a digital health intervention, which included a smartphone app, smartwatch, and blood pressure monitor, for patients hospitalized with acute myocardial infarction found a substantially lower three-day readmission rate in the intervention group (6.5%) compared to the control group (16.8%). After adjusting for confounding variables, the intervention group showed a 52% lower risk of all-cause 30-day readmission [21]. 

However, not all digital tools have shown positive results. Emery et al. evaluated a voiceover PowerPoint presentation designed to educate patients on catheter care and found no significant reduction in either all-cause readmissions or catheter-related bloodstream infections [17]. In another study that compared discharge instructions delivered by telephone, text message, and written summaries, no statistically significant difference was found in emergency department revisits within one week across the different methods [20]. These findings suggest that while video-assisted and digital discharge interventions can positively impact patient outcomes, particularly in reducing readmissions, their success may depend on the specific format, context of use, and patient population.

Comprehension and Patient Engagement

Improving patient understanding and engagement is a central objective of discharge interventions, and several studies indicate that digital and video-based tools may contribute positively to these outcomes. For example, a single-centre trial found that patients who viewed online videos of their discharge instructions demonstrated better comprehension of both their diagnosis and their post-discharge care plan [15]. Similarly, in a randomized controlled trial involving patients undergoing hip replacement surgery, those who received video-assisted discharge education reported higher levels of satisfaction with their care experience [27]. Conversely, in a study by Shuen et al., no significant differences in satisfaction were observed among patients and caregivers who received discharge instructions via text message, phone call, or traditional methods [20]. This suggests that the format of discharge communication may not universally impact satisfaction levels.

Further evidence of the benefits of digital tools comes from a study that assessed the use of a smartphone-based application offering live health coaching after percutaneous coronary intervention. Patients who used the app showed higher rates of participation in cardiac rehabilitation and were more likely to attend their one-month follow-up appointments compared to historical controls [25]. This illustrates the potential of digital interventions to enhance patient engagement and improve adherence to post-discharge care plans. Despite these promising outcomes, it is important to consider that selection bias may play a role in the reported benefits. Patients who choose to participate in digital programs are often more proactive and health-literate, which could influence the observed improvements in comprehension and engagement.

Cost Reduction and Disease Control

Digital interventions have also demonstrated benefits in disease control and the reduction of healthcare costs. In a randomized controlled trial focused on diabetes management, patients who received mobile health coaching achieved a 0.6% greater reduction in glycated hemoglobin (HbA1c) levels compared to those receiving usual care, highlighting the potential for such tools to improve chronic disease outcomes [23]. Similarly, Rosner et al. evaluated an automated digital patient engagement platform and found that 93% of patients in the intervention group achieved a target cost of $0, compared to 84% in the baseline group. The same study reported a 45.4% relative reduction in 90-day readmission rates, indicating significant cost savings and improvements in post-discharge outcomes [24]. Further supporting the role of digital tools in chronic disease management, another study found that video-based discharge education enhanced patients’ self-efficacy in handling medication-related challenges, reinforcing the value of these tools in promoting better self-management practices [29].

Taken together, these findings underscore the potential of video-assisted and digital information systems to enhance continuity of care for elderly patients. By reducing readmissions and revisit rates, improving patient comprehension and engagement, and lowering healthcare costs, these interventions represent a promising avenue for improving post-discharge outcomes. Nevertheless, further research is necessary to address potential selection biases and to identify the most effective and scalable strategies for implementation across diverse patient populations.

Satisfaction of Patients and Caregivers

Pertaining to the existing evidence on the value of video-assisted and digital information systems for post-discharge care of elderly patients, from the perspectives of patients and carers, there is a growing body of proof evaluating patient and caregiver satisfaction with video-assisted and digital discharge tools, with findings generally indicating favorable responses. These studies suggest that such tools can enhance the discharge experience, particularly when they are personalized and clearly communicated. Bloch and Bloch examined the use of video discharge instructions as a supplement to standard verbal and written formats in a pediatric emergency department setting. While the study focused on a pediatric population, its relevance extends to geriatric care due to similarities in caregiver involvement across both groups. The results demonstrated that short video instructions significantly improved caregiver knowledge and satisfaction compared to conventional discharge methods [28]. In a digital health intervention study involving patients hospitalized for acute myocardial infarction, post-discharge app usability scores averaged approximately 58 out of 100, indicating moderate satisfaction with the platform. While overall feedback was positive, the study highlighted a need for further refinement in user interface design and platform functionality to enhance the patient and caregiver experience [21]. The Care TV study introduced a personalized, interdisciplinary audiovisual discharge summary and reported high patient satisfaction. Many participants found the tailored information clear and useful, with over half describing the intervention as "very helpful" and a "good idea." The structured communication, often perceived as “hearing it from the horse’s mouth”, was credited with improving understanding and recall of discharge instructions [26].

In contrast, a study by Shuen et al. found no statistically significant differences in satisfaction among patients receiving discharge information via text messages, phone calls, or written summaries, suggesting that the mode of delivery alone may not be sufficient to enhance patient experience [20]. Another study evaluating video discharge instructions for patients with mild traumatic brain injury found no difference in the severity of post-concussion symptoms across groups. However, those who received video instructions reported higher satisfaction with their discharge education and experienced fewer symptoms of anxiety and depression, indicating potential psychological benefits associated with enhanced communication [21]. Similarly, Heyworth et al. implemented a web-based portal designed for medication reconciliation following hospital discharge. The intervention was well received, with more than 90% of participants indicating a willingness to use the portal again, reflecting strong acceptance of digital tools for managing post-discharge care and improving care coordination [14].

These studies suggest that patients and caregivers are generally receptive to video-assisted and digital discharge instructions. Potential selection bias should be considered, as participants in these studies often exhibit higher baseline engagement with digital technologies. Notably, interventions that provided personalized, structured, and accessible discharge summaries consistently reported the highest levels of satisfaction. These findings underscore the importance of tailoring digital discharge tools to individual patient needs and communication preferences to optimize their impact.

Lack of Exposure to Technology

Throwing light on the challenges related to the use of video-assisted and digital information systems for post-discharge care of elderly patients from the perspectives of patients and carers is the study by Alexander et al. [18] that explored patient preferences regarding the use of technology to communicate symptoms following hospital discharge. The study found that while many patients were open to using technology to discuss symptoms perceived as minor or of low concern, face-to-face communication remained the preferred method when addressing more serious health issues. Among the various technological options assessed, online discussion forums were the least favored by participants. The study also highlighted notable demographic differences: patients aged 66 to 80 reported the lowest levels of comfort with using technology for healthcare communication. In contrast, individuals from higher-income households were more inclined to prefer telephone or technology-based interactions over in-person visits, suggesting that socioeconomic status may influence receptivity to digital health tools.

Socioeconomic Status

Many studies investigating the use of technology in healthcare have systematically excluded patients who were not fluent in the study’s primary language, most often English, as seen in multiple investigations [16, 27, 30]. While this exclusion criterion was intended to minimize the risk of miscommunication and ensure data accuracy, it inadvertently led to the underrepresentation of individuals from lower socioeconomic backgrounds. These populations, who are more likely to face language barriers, were also found to have lower digital literacy, making them less likely to enroll in or benefit from digital health initiatives. Taken together, the evidence suggests that older adults (aged 66 to 80 years) and individuals from lower socioeconomic groups are among the least likely to adopt or feel comfortable with technology-based communication and instruction following hospital discharge. This highlights the need for more inclusive design and implementation strategies that consider linguistic diversity, digital literacy, and socioeconomic disparities to ensure equitable access to post-discharge care innovations.

Physician Factors

Shedding light on the challenges related to the use of video-assisted and digital information systems in healthcare, from the perspective of healthcare workers, Okoniewska et al. [30] identified several critical barriers to achieving effective and timely discharge in acute care settings. Among the most prominent challenges were poor communication among care team members, ambiguous role definitions, and a lack of adequate resources. Healthcare providers in the study underscored the importance of implementing structured discharge processes, such as discharge rounds, clear and consistent documentation, and the use of concise communication formats like bullet points, to improve the quality and efficiency of care transitions. Similarly, Eren et al. [27] examined the challenges and enablers of caregiver training in home healthcare. Their findings revealed that inadequate team communication, the absence of comprehensive training materials, and the provision of incomplete or inaccurate discharge information significantly hindered effective caregiver preparation. These studies collectively highlight the need for well-organized, transparent, and resource-supported discharge processes to ensure both patients and caregivers are adequately equipped for the transition from hospital to home.

Nursing and Occupational Therapy Perspectives

A pilot study evaluating a video-based education intervention for discharge planning found that nurses responded positively to the initiative. They noted that the intervention improved the discharge process by allowing patients more time to absorb information, reflect on their care instructions, and ask questions prior to leaving the hospital [29]. This additional time for patient engagement was seen as a valuable enhancement to traditional discharge planning practices. In a related investigation, Wong et al. [32] identified key barriers to effective discharge planning and grouped them into four domains: system factors, healthcare professional factors, patient factors, and social factors. Systemic issues such as high staff turnover and the tendency toward premature discharges were highlighted as particularly problematic. The study also revealed disparities in how various healthcare professionals addressed patient needs; physicians were found to sometimes overlook patients' social circumstances, whereas nurses and occupational therapists demonstrated a greater attentiveness to these aspects. To address these challenges, the authors recommended appointing a dedicated care transition leader responsible for overseeing the discharge process and ensuring that essential information is effectively communicated to the primary family caregiver.

Social Factors

Social factors such as transportation availability, living conditions, and the overall ease of transitioning from hospital to home play a crucial role in discharge planning. Elements like family support, the presence and availability of caregivers, and the work schedules of key stakeholders often determine not only the timing of discharge but also the success of the care transition process [31]. Nurses and occupational therapists, in particular, have been consistently recognized for their ability to assess and address these social determinants effectively, ensuring that patients are adequately prepared for discharge and supported in the post-hospital environment [32].

Limitations

This review is subject to several limitations. One of the primary concerns is the heterogeneity of the digital interventions evaluated, which range from smartphone applications and video-assisted discharge instructions to broader digital discharge summary systems. Such variation in intervention types may limit the comparability of findings across studies and reduce the ability to draw definitive conclusions about the effectiveness of any single approach. Another key limitation is the potential for selection bias. Most studies included in the review relied on voluntary participation in digital health programs, thereby favoring individuals who were already comfortable with technology. This tendency disproportionately represents patients with higher digital literacy and socioeconomic status, which may undermine the generalizability of the findings to more vulnerable populations who are less likely to engage with such tools. A single author performed the initial screening process. This approach, while efficient, introduces a potential for bias in the selection of studies for inclusion in the review.

In addition, the inclusion of studies involving pediatric patients, based on parallels with geriatric care due to caregiver involvement, may not be entirely appropriate. The discharge education needs of pediatric populations, typically mediated through parents or guardians, differ in important ways from those of elderly patients, particularly those requiring cognitive, emotional, or physical support. The review also fell short in addressing the unique needs of specific subpopulations, such as individuals with Alzheimer’s disease, bedridden patients, those dependent on primary caregivers, and residents of long-term care or assisted living facilities. Each of these groups faces distinct challenges that merit individualized consideration and tailored intervention strategies. Lastly, the inclusion of both qualitative and quantitative studies may have introduced variability in study designs, outcome measures, and analytical frameworks. This methodological diversity complicates the synthesis of findings and may affect the consistency and interpretability of the overall conclusions.

## Conclusions

This review underscores the importance of timely and well-coordinated discharge planning, emphasizing that effective care transitions should begin well in advance of a patient’s discharge. Allowing patients adequate time to review discharge information, raise questions, and clarify uncertainties is a critical component of ensuring comprehension and confidence in post-discharge care. While many patients responded positively to digital health interventions, studies that incorporated a more holistic approach to discharge planning tended to achieve better outcomes. Interventions that combined digital tools with personalized support and clear communication were notably more effective in enhancing continuity of care. Video-based discharge education demonstrated particular promise in improving patient understanding and satisfaction. However, older adults were generally less likely to adopt these digital solutions, often due to limited digital literacy, cognitive or sensory impairments, and a range of social determinants. These barriers were especially evident among patients from lower socioeconomic backgrounds, where access to technology and familiarity with digital tools may be limited.

Resistance to digital health solutions was not confined to patients. Among healthcare professionals, physicians were identified as the group most resistant to adopting digital discharge systems, often due to workflow disruptions, perceived inefficiencies, or skepticism about the value of such tools. In conclusion, while video-assisted discharge education and digital information systems offer considerable potential to improve post-discharge care for older adults, their success hinges on multiple factors. These include the quality and timing of discharge planning, the digital readiness of patients, and the willingness of a multidisciplinary healthcare team to support and integrate these technologies into standard practice.

Overall, while digital and video-assisted discharge systems represent valuable innovations in post-discharge care, their success depends on thoughtful implementation. This includes addressing technological, social, and systemic barriers, fostering collaboration among healthcare providers, and ensuring accessibility and usability for diverse patient populations. Future research should focus on optimizing these tools for vulnerable groups and exploring scalable models that promote equitable access to digital health solutions.
